# Complete Chloroplast Genomes from *Sanguisorba*: Identity and Variation Among Four Species

**DOI:** 10.3390/molecules23092137

**Published:** 2018-08-24

**Authors:** Xiang-Xiao Meng, Yan-Fang Xian, Li Xiang, Dong Zhang, Yu-Hua Shi, Ming-Li Wu, Gang-Qiang Dong, Siu-Po Ip, Zhi-Xiu Lin, Lan Wu, Wei Sun

**Affiliations:** 1 Key Laboratory of Beijing for Identification and Safety Evaluation of Chinese Medicine, Institute of Chinese Materia Medica, China Academy of Chinese Medical Sciences, Beijing 100700, China; xxmeng@icmm.ac.cn (X.-X.M.); lxiang@icmm.ac.cn (L.X.); dzhang1987@icmm.ac.cn (D.Z.); yhshi@icmm.ac.cn (Y.-H.S.); justuswu@163.com (M.-L.W.); 2 School of Chinese Medicine, Faculty of Medicine, The Chinese University of Hong Kong, Shatin 999077, N.T., Hong Kong, China; lisaxian@cuhk.edu.hk (Y.-F.X.); paulip@cuhk.edu.hk (S.-P.I.); linzx@cuhk.edu.hk (Z.-X.L.); 3 Amway (China) Botanical Research and Development Center, Wuxi 214145, China; tony.dong@Amway.com

**Keywords:** *Sanguisorba*, chloroplast genome, molecular structure, phylogenetic analysis

## Abstract

The genus *Sanguisorba*, which contains about 30 species around the world and seven species in China, is the source of the medicinal plant *Sanguisorba officinalis*, which is commonly used as a hemostatic agent as well as to treat burns and scalds. Here we report the complete chloroplast (cp) genome sequences of four *Sanguisorba* species (*S. officinalis*, *S. filiformis*, *S. stipulata*, and *S. tenuifolia* var. *alba*). These four *Sanguisorba* cp genomes exhibit typical quadripartite and circular structures, and are 154,282 to 155,479 bp in length, consisting of large single-copy regions (LSC; 84,405–85,557 bp), small single-copy regions (SSC; 18,550–18,768 bp), and a pair of inverted repeats (IRs; 25,576–25,615 bp). The average GC content was ~37.24%. The four *Sanguisorba* cp genomes harbored 112 different genes arranged in the same order; these identical sections include 78 protein-coding genes, 30 tRNA genes, and four rRNA genes, if duplicated genes in IR regions are counted only once. A total of 39–53 long repeats and 79–91 simple sequence repeats (SSRs) were identified in the four *Sanguisorba* cp genomes, which provides opportunities for future studies of the population genetics of *Sanguisorba* medicinal plants. A phylogenetic analysis using the maximum parsimony (MP) method strongly supports a close relationship between *S. officinalis* and *S. tenuifolia* var. *alba*, followed by *S. stipulata*, and finally *S. filiformis*. The availability of these cp genomes provides valuable genetic information for future studies of *Sanguisorba* identification and provides insights into the evolution of the genus *Sanguisorba*.

## 1. Introduction

The genus *Sanguisorba* belongs to the Rosaceae; there are about 30 species in the genus *Sanguisorba* in the world, mainly distributed in Asia, Europe, and North America (eFlora of China: http://www.eflora.cn/). There are seven species and six varieties of *Sanguisorba* in China [1], distributed in both northern and southern China, especially in the northeast provinces. *Sanguisorba officinalis* has been recorded as a medicinal plant that is commonly used to treat water and fire burns, hemorrhoidal bleeding, and hematochezia [2]. Diyu Shengbai Tablet, a Chinese patent medicine, is mainly composed of *S. officinalis*, and contains active chemical components including saponins, flavonoids and tannins [3]. It can protect the hematopoietic system, elevate the peripheral blood white blood cells, neutrophils, and platelets, improve bone marrow micro-circulation, and adjust and improve body immunity and other functions. It is also often clinically used as an adjuvant during chemotherapy [3].

The chloroplast genome is ~100–150 kb in length and contains a wealth of evolutionary information, which can be used to reveal phylogenetic relationships among closely related species and can also be valuable for species identification [4,5]. It has been widely used in species identification, phylogenetic evolution, and genetic engineering-related research [6,7]. With the rapid development of high-throughput sequencing technologies and bioinformatics tools, the cost of sequencing chloroplast genome has been significantly reduced, making the large-scale acquisition of chloroplast genomic sequences possible [8,9]. This has made possible the study of chloroplast genomes in terms of population genetic structure, phylogenetic evolution, and species identification.

However, molecular research on the genus *Sanguisorba* is still very scarce. Currently, there are no reports on the chloroplast genome sequence of the genus *Sanguisorba*, which seriously hampers molecular identification, phylogenetic, genetic, and breeding research involving the genus. In this study, we report the chloroplast genome assembly, annotation, and structural analysis of four *Sanguisorba* species (*S. officinalis*, *S. filiformis*, *S. stipulata*, and *S. tenuifolia* var. *alba*) as well as the complete chloroplast genome sequences of these species, which are the first four sequenced members of the genus *Sanguisorba.* In addition, we compared the chloroplast genomes of the four *Sanguisorba* species in detail (e.g., based on IR expansion/contraction and difference regions). From this we constructed a phylogenetic tree using the maximum parsimony (MP) method based on both the whole cp genome and on common protein-coding genes, respectively. Overall, our results provide useful genetic information on the chloroplast of *Sanguisorba* species, as well as their relative position in phylogenetic tree.

## 2. Results and Discussion

### 2.1. Chloroplast Genome Assembly and Features

**Figure 1:**
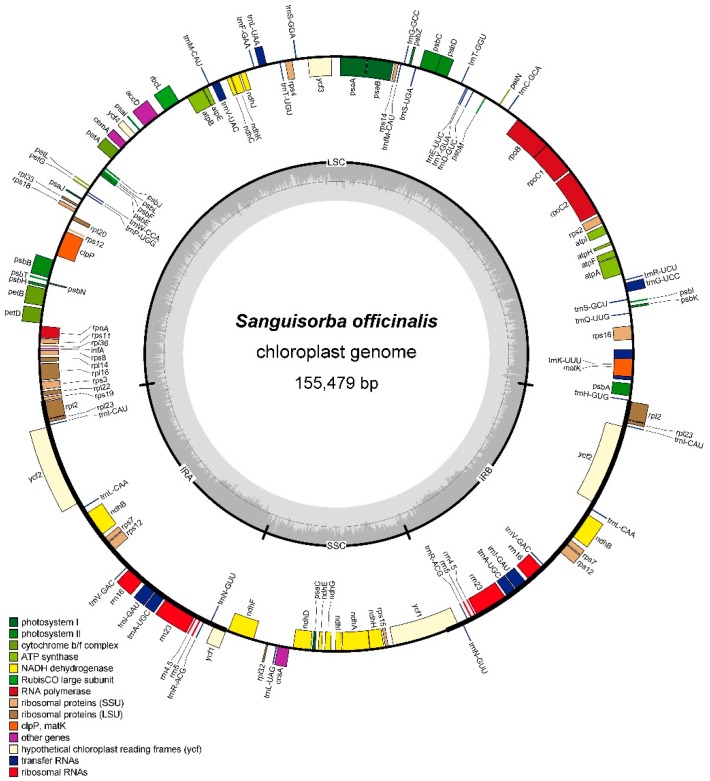
Gene map of *Sanguisorba officinalis* chloroplast genome. Genes shown inside the circle are transcribed clockwise, and those outside are counterclockwise. Genes in different functional groups are color-coded.

Using an Illumina HiSeq X platform, four *Sanguisorba* species were sequenced to produce 11,554,422–18,828,898 paired-end raw reads. After screening these paired-end reads, 598,166 to 1,080,144 cp genome reads were successfully mapped with 569X to 1032X sequencing depth (Table 1). In this study, the sequencing depth was high enough to satisfy the technical requirements of an organelle genome assembly. In total, the complete cp genomes of the four *Sanguisorba* species were similar in length, ranging from 155,127 bp (*S. stipulata*) to 155,479 bp (*S. officinalis*) (Figure 1 and Figures S1–S3, and Table 1), with the typical quadripartite structure of angiosperms. All four cp genomes contained a large single-copy regions (LSC, 84,405–85,557 bp) and a small single-copy regions (SSC, 18,550–18,768bp), separated by a pair of inverted repeats regions (IRs, 25,576–25,615 bp). 

The average GC content of the four *Sanguisorba* cp genomes was ~37.23%; in this respect they showed only minor differences from one another and resembled the cp genomes of other reported Rosaceae species [10,11,12]. Nevertheless, the GC content is unevenly distributed in the four *Sanguisorba* cp genomes. The GC content of the IR regions (~42.7%) is significantly higher than in the LSC region (~35.3%) or the SSC regions (~31.3%). We speculate that this may be a reason for the divergence of the conservation between the IR and SC regions [8,13].

All four *Sanguisorba* cp genomes possessed 112 unique genes including 78 protein-coding genes, 30 tRNA genes, and four rRNA genes (Table 2). Of these, six protein-coding genes, seven tRNA genes, and four rRNA genes are duplicated in the IR regions, making a total of 129 genes shared (Table 2). Our results showed that the four *Sanguisorba* cp genomes were highly conserved in gene type, order, and content. We classified the 112 genes into different categories according to their function, and the details are shown in Table 2. In addition, two pseudogenes (*ycf1* and *infA*) were found in the four cp genomes. There were 18 genes located in the IR regions as follows: *rrn16*, *rrn23*, *rrn5*, *rrn4.5*, *trnA-UGC*, *trnI-CAU*, *trnI-GAU*, *trnL-CAA*, *trnN-GUU*, *trnR-ACG*, *trnV-GAC*, *rps7*, *rps12*, *rpl2*, *rpl23*, *ndhB*, *ycf1*, *and ycf2* (Figure 1 and Figures S1–S3). *rps12* is a trans-spliced gene, in which two 3’ end residues are located in the IR region and the 5’ end in the LSC region (Figure 1 and Figures S1–S3). This is a common phenomenon in the cp genomes of higher plants [14,15]. Significantly, the *ycf15* gene is located in cp genome of most angiosperm while is absent from the *Sanguisorba* cp genomes. This phenomenon was also found to occur in *Cedrela odorata* [7], *Schisandra chinensis* [8], *Cremastra appendiculata* [16] and *Aristolochia debilis* [17].

Introns play an important role in the regulation of alternative gene splicing [18,19]. We found that 17 genes contained introns in all four *Sanguisorba* cp genomes, of which 11 are protein-coding genes and six are tRNA genes. 14 of the 17 contain a single intron, whereas three (*clpP*, *rps12*, and *ycf3*) have two introns. The largest intron, located into the *trnK-UUU* gene, ranged 2508 bp to 2516 bp in the four species (Table 3 and Tables S1–S3). The *matK* gene is located in the intron of *trnK-UUU* gene.

### 2.2. Codon Usage

The total length of the protein coding genes from the four *Sanguisorba* cp genomes is 78,582~78,612 bp, and these genes are encoded by 22,760~22,768 codons. Protein coding genes thus accounted for 50.6~50.9% of the whole genome sequence. The most frequent amino acid is leucine, with 2387~2400 (10.5%) of the codons, but cysteine is the least frequent in the four *Sanguisorba* cp genomes, with only 260~262 (1.1%) of all codons. Within the protein-coding sequences (CDS), the AT content of codons at the first to third positions is 54.5%, 61.9~62.0%, and 69.5~69.6%, respectively. The fact is that the AT content of the codons is the highest with the third position, and it’s common in land plants [7,13,20,21]. The same phenomenon was also found in the frequency of codon usage. All preferred synonymous codons (RSCU > 1) ended with A or U except the codons of *trnL-CAA*; however, most non-preferred synonymous codons (RSCU < 1) ended with G or C (Table 4 and Table S4–S6).

### 2.3. Long Repeats and SSR Analysis

For long repeats analysis, the four cp genomes enclose long repeats with a total number ranging from 39 to 53 with at least 30 bp per repeat unit. Taking *S. officinalis* as an example, a number of 49 repeats were detected. These included 24 palindromic repeats, 17 forward repeats, six reverse repeats, and two complement repeats. Most repeats showed lengths between 30 and 44 bp and are in intergenic regions or intron sequences.

SSRs, also called as microsatellites, are tandemly repeated sequences that consist of 1–6 nucleotide repeat units. SSRs are widely distributed in cp genomes in general and are important for studies of plant populations. Because of their high level of polymorphism, SSRs are widely used as molecular markers for species authentication, molecular breeding, and population genetics [22,23,24,25]. Here, we identified many SSRs in the cp genomes, ranging from 79 in *S. tenuifolia* var. *alba* to 91 in *S. stipulata*. Most of the SSRs are mononucleotide repeats, whose amount ranges from 55 (*S. tenuifolia* var. *alba*) to 69 (*S. stipulata*). The number of di-, tri-, tetra-, penta-, and hexanucleotide repeats found was 9~12, 3~4, 7~9, 0~1, and 1~2, respectively (Table 5). Most of the mononucleotide SSRs belonged to the A/T type in the four *Sanguisorba* species. The highest number of SSRs found was in *S. stipulata*, which showed 68 of 69 identified mononucleotide SSRs. The lowest number of SSRs found was 55 of the 59 found in *S. officinalis*. These results are consistent with those of previous studies that found that polyadenine (polyA) and polythymine (polyT) content were higher than polyguanine (polyG) and polycytosine (polyC) content in the cpSSRs of many plants [26]. We speculate that the abundance of A/T SSRs may be associated with the AT richness of these cp genomes [13,27].

### 2.4. IR Contraction and Expansion

**Figure 2:**
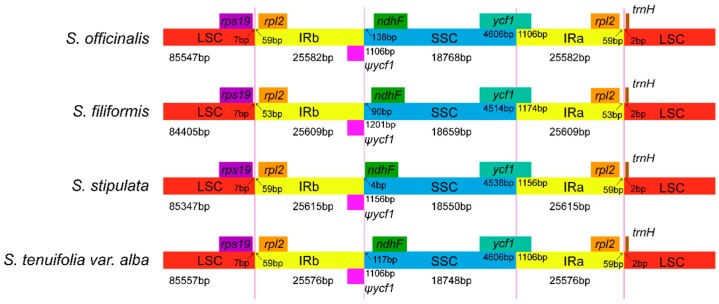
Comparison of the border regions of the LSC, SSC, and IR among four chloroplast genomes. Ψ: pseudogenes.

It is well known that IRs are the most conserved regions in chloroplast genomes, and the contraction and expansion at the borders of IR regions are common evolutionary events. It is also a main cause of length variation in the chloroplast genomes [28,29]. In this study, we compared the IR/SSC and IR/LSC boundaries of the four *Sanguisorba* cp genomes (Figure 2). In the four *Sanguisorba* species, the IRb/SSC boundary extends into functional *ycf1* genes, yielding a pseudogene *ycf1*, which have a length of 1106~1201 bp in the four species. A previous study reported that the pseudogene *ycf1* may be useful for researching variation among cp genomes in higher plants or algae [30]. In addition, we found no overlap between the *ycf1* pseudogene and *ndhF* in the four species. The *ndhF* gene is found in the SSC region, and was 138 bp, 90 bp, four bp, and 117 bp away from the IRb/SSC boundary in *S. officinalis*, *S. filiformis*, *S. stipulata*, and *S. tenuifolia* var. *alba*, respectively. The *trnH* gene was found in the same position of the same LSC region in the four species, which is only two bp away from the IRb/SSC boundary. In the cp genome, variation in the IR/SSC and IR/LSC boundaries is governed by a dynamic and random process that is confined to conservative expansions and contractions [31,32]. There are many studies about the mechanisms responsible for IR expansion, and the leading view is that short IR expansions could be caused by gene conversion, but large IR expansions may be the result of double-strand DNA break repair (DSBR) [33,34]. In contrast, there are few reports on the mechanisms of IR contraction. However, Peery et al. proposed that DSBR theory was not only the main mechanism of IR region expansion, but also the main mechanism of IR region contraction [35].

### 2.5. Comparative Chloroplast Genomic Analysis

**Figure 3:**
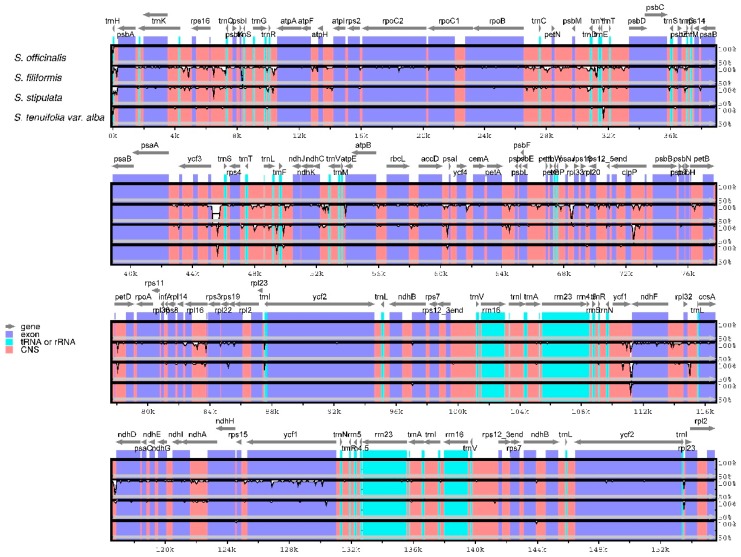
Comparison of the four *Sanguisorba* chloroplast genomes using mVISTA. CNS indicates conserved noncoding sequences. The Y-scale represents the percent identity between 50% and 100%.

With the annotated *S. officinalis* cp genome as a reference, the whole cp genome of the four *Sanguisorba* species were compared and drawn by mVISTA to show sequence divergence (Figure 3), which is important for further phylogenetic analyses and species identification. Comparative genome analysis found that there is a high similarity between the cp genomes of all *Sanguisorba* species. The LSC and SSC regions are more divergent than the two IR regions, which is common in other higher plants and may be due to copy corrections between two IR regions by gene conversion [36]. Moreover, the coding regions have less variability proportions than the non-coding regions. The highest divergence among the four *Sanguisorba* cp genomes occurs in the intergenic spacers region, which contains *trnE*-*trnT*, *trnS*-*psbZ*, *trnS*-*ycf3*, *trnF*-*ndhJ*, *accD*-*psal*, and *ycf1*-*ndhF*. In this study, we found that the more conserved coding regions are the four rRNA located in IR region.

### 2.6. Phylogenetic Analysis

**Figure 4:**
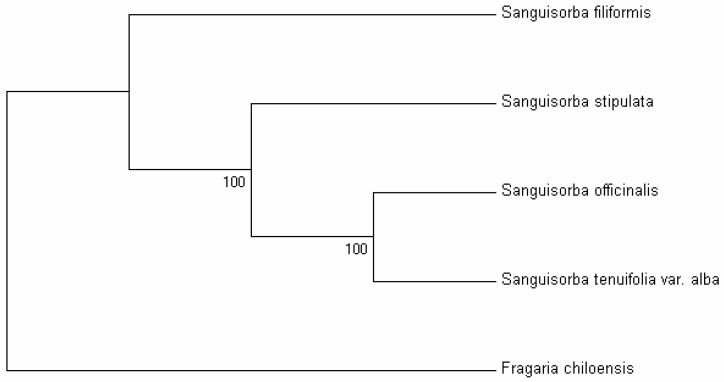
Phylogenetic relationships between the four *Sanguisorba* species determined by whole cp genome sequences using the maximum parsimony (MP) method. *Fragaria chiloensis* was set as the outgroup.

Chloroplast genomes contain abundant genetic information that is widely applied in plant identification and phylogenetic studies [6,37,38,39]. *Sanguisorba* belongs to the subfamily Rosoideae in the Rosaceae. Previous studies have reported phylogenetic relationships within the Rosaceae that were analyzed based on chloroplast regions [40,41]. Here, the availability of the completed cp genomes and protein coding genes of the four *Sanguisorba* species provide us with sequence and gene information for studying the molecular evolution and phylogeny of the genus *Sanguisorba* [9,42]. In this study, two datasets (i.e., the whole complete cp genome and the set of protein coding genes) from the cp genomes of the four *Sanguisorba* species and one outgroup (*Fragaria chiloensis*) were used to perform phylogenetic analysis. Phylogenetic trees were generated using the maximum parsimony (MP) method based on two datasets with the same topologies (Figure 4 and Figure S4). For the four *Sanguisorba* species, *S. officinalis* has the closest relationship with *S. tenuifolia* var. *alba*, followed by *S. stipulata*, and has the least close relationship with the *S. filiformis*. In addition, both *S. stipulata* and *S. filiformis* group into a monophyletic clade.

## 3. Materials and Methods

### 3.1. Plant Materials and DNA Extraction

Fresh leaves of four *Sanguisorba* species were collected from Jilin and Yunan Provinces in China. Then we washed the leaves powder with HF buffer (100 mmol·L^−1^ Tris-HCl pH 8.0, 20 mmol·L^−1^ EDTA, 0.7 mol·L^−1^ NaCl, 2% PVP, and 0.2% 2-mercaptoethanol). HF buffer (600 μL) was added to leaves powder (~100 mg), the mixture vortexed vigorously for 3 min, centrifuged for 5 min at 12,000 rpm, and the supernatant discarded. Finally the total genomic DNA of each sample was isolated from the leaves powder by Plant Genomic DNA Kits (Tiangen Biotech Co., Beijing, China), according to the manufacturer’s instructions. The DNA quality and quantity of each sample was estimated by a NanoDrop 2000 Spectrophotometer (Nanodrop Technologies, Wilmington, DE, USA) and a Qubit3.0 Fluorometer (Thermo Scientific, Waltham, MA, USA), as well as by agarose gel electrophoresis.

### 3.2. Chloroplast Genome Sequencing, Assembly and Annotation

After DNA was purified and prepared, ~2 μg was used to construct shotgun libraries. Genomic DNA was taken and sheared into 450 bp contigs with the Covaris M220 Focused-ultrasonicator (Covaris, Woburn, MA, USA). The library was constructed by TruSeq^TM^ DNA Sample Prep Kit (Illumina Inc., San Diego, CA, USA), according to the manufacturer’s instructions. An Illumina HiSeq X platform was used for sequencing. Clean reads were obtained by using the Fastqc trim tool [43]. We then extracted cp-like reads from trimmed reads by performing BLASTs [44] using reference sequences (*Rosa roxburghii*, accession No.: NC_032038). Sequence assembly was performed by using SOAPdenovo [45], and the contigs were aligned using SSPACE [46]. The complete chloroplast genomes of the four *Sanguisorba* species were annotated using the CpGAVAS web service [47]. The tRNA genes were confirmed using tRNAscan-SE [48,49]. OGDRAW software (http://ogdraw.mpimp-golm.mpg.de/) [50] was used to draw circular cp genome maps for each species. The validated complete cp genome of the four *Sanguisorba* species were deposited in GenBank (https://www.ncbi.nlm.nih.gov/): *S. officinalis*, MF678801; *S. filiformis*, MF678800; *S. stipulata*, MF678798; and *S. tenuifolia* var. *alba*, MF678799.

### 3.3. Genome Comparison and Structural Analyses

The IR and SC boundary regions of the four *Sanguisorba* species were compared and examined. Comparison of the four cp genomes was performed using the Shuffle-LAGAN mode in mVISTA [51,52], with the annotation of *S. officinalis* used as the reference. In addition, we analyzed the codon usage, relative synonymous codon usage values (RSCU), and GC content using MEGA5 [53]. SSRs were identified by MISA (http://pgrc.ipk-gatersleben.de/misa/) [54] with minimum repeat numbers of 10, 5, 4, 3, 3, and 3 for mono-, di-, tri-, tetra-, penta-, and hexanucleotides, respectively. The forward and inverted repeats in the *Sanguisorba* cp genome were detected using REPuter [55] with a minimal repeat sequence of 30 bp and a sequence identity of 90%.

### 3.4. Phylogenetic Analyses

Phylogenetic analyses were performed for the four *Sanguisorba* species using *Fragaria chiloensis* (Rosaceae) as an outgroup. The complete cp genome sequences and protein coding genes shared in four *Sanguisorba* species and *Fragaria chiloensis* (accession No.: NC_019601) [56] were aligned by ClustalW2 [57]. Phylogenetic trees were constructed using the maximum parsimony (MP) method in PAUP*4.0b10 [58]. A heuristic search was performed using the MULPARS option, with the random stepwise addition of sequences in 1000 replications and tree bisection reconnection (TBR) branch swapping. The branch support of the phylogenetic tree was 1000 bootstrap replicates.

## 4. Conclusions

The complete cp genome sequences of four *Sanguisorba* species (*S. officinalis*, *S. filiformis*, *S. stipulata*, and *S. tenuifolia* var. *alba*), the first four sequenced members of the genus *Sanguisorba*, were assembled, annotated and analyzed in this study. The genome structure, gene content, and gene order were similar in the four species. Long repeats and SSRs reported here provide opportunities for the development of new molecular markers to study medicinal plants in the genus *Sanguisorba*. Phylogenetic analysis strongly supported that *S. officinalis* has the closest relationship with *S. tenuifolia* var. *alba*, followed by *S. stipulata*, and then *S. filiformis*. The available genome data presented in this paper provides a basis for further research on the evolution of the genus *Sanguisorba*, as well as for species identification.

## Figures and Tables

**Table 1:** Sequence information and Illumina next-generation sequencing (NGS) data of the four *Sanguisorba* chloroplast genomes.

Species	Raw Reads No.	Mapped Reads No.	Sequencing Depth	Cp Genome Length (bp)	GC Content (%)	LSC ^a^ (bp)	SSC ^a^ (bp)	IRs ^a^ (bp)
*S. officinalis*	11,554,422	609,666	581X	155,479	37.19	85,547	18,768	25,582
*S. filiformis*	16,876,554	656,271	628X	154,282	37.33	84,405	18,659	25,609
*S. stipulata*	18,828,898	1,080,144	1032X	155,127	37.23	85,347	18,550	25,615
*S. tenuifolia* var. *alba*	18,366,336	598,166	569X	155,457	37.20	85,557	18,748	25,576

^a^ LSC (large single-copy regions), SSC (small single-copy regions), and IRs (inverted repeats regions).

**Table 2:** List of genes encoded by the four *Sanguisorba* chloroplast genomes.

Category	Group	Name
Self-replication	rRNA genes	*rrn4.5a*, *rrn5a*, *rrn16a*, *rrn23a*
	tRNA genes	*trnA-UGC* *^,a^, *trnC-GCA*, *trnD-GUC*, *trnE-UUC*, *trnF-GAA*, *trnfM-CAU*, *trnG-GCC*, *trnG-UCC* *, *trnH-GUG*, *trnI-CAU* ^a^
		*trnI-GAU* *^,a^, *trnK-UUU* *, *trnL-CAA* ^a^, *trnL-UAA* *, *trnL-UAG*, *trnM-CAU*, *trnN-GUU* ^a^, *trnP-UGG*, *trnQ-UUG*, *trnR-ACG* ^a^, *trnR-UCU*, *trnS-GCU*, *trnS-GGA*, *trnS-UGA*, *trnT-GGU*, *trnT-UGU*, *trnV-GAC* ^a^, *trnV-UAC* *, *trnW-CCA*, *trnY-GUA*
	Small subunit of ribosome	*rps2*, *rps3*, *rps4*, *rps7*^a^, *rps8*, *rps11*, *rps12* **^,a^, *rps14 rps15*, *rps16* *, *rps18*, *rps19*
	Large subunit of ribosome	*rpl2* *^,a^, *rpl14*, *rpl16* *, *rpl20*, *rpl22*, *rpl23* ^a^, *rpl32*, *rpl33*, *rpl36*
	DNA dependent RNA polymerase	*rpoA*, *rpoB*, *rpoC1* *, *rpoC2*
Genes for phytosynthesis	Subunits of NADH-dehydrogenase	*ndhA* *, *ndhB* *^,a^, *ndhC*, *ndhD*, *ndhE*, *ndhF*, *ndhG*, *ndhH ndhI*, *ndhJ*, *ndhK*
	Subunits of photosystem I	*psaA*, *psaB*, *psaC*, *psaI*, *psaJ*
	Subunits of photosystem II	*psbA*, *psbB*, *psbC*, *psbD*, *psbE*, *psbF*, *psbH*, *psbI*, *psbJ*, *psbK*, *psbL*, *psbM*, *psbN*, *psbT*, *psbZ*
	Subunits of cytochrome b/f complex	*petA*, *petB* *, *petD* *, *petG*, *petL*, *petN*
	Subunits of ATP synthase	*atpA*, *atpB*, *atpE*, *atpF*, *atpH*, *atpl*
	Large subunit of RuBisCO	*rbcL*
Other genes	Maturase	*matK*
	Envelope membrane protein	*cemA*
	Subunit of Acetyl-CoA-carboxylase	*accD*
	C-type cytochrome synthesis gene	*ccsA*
	Protease	*clpP* **
Genes of unknown function	Open Reading Frames (ORF, ycf)	*ycf1*, *ycf2a*, *ycf3* **, *ycf4*
	Pseudo genes	*ycf1*, *infA*

* Gene with one intron, ** Gene with two introns, ^a^ Gene with two copies.

**Table 3:** The length of exons and introns in genes with introns in the *Sanguisorba officinalis* chloroplast genome.

No.	Gene	Location	Exon I (bp)	Intron I (bp)	Exon II (bp)	Intron I (bp)	Exon III (bp)
1	*clpP*	LSC	69	938	291	658	228
2	*ndhA*	SSC	563	1185	541		
3	*ndhB*	IR	777	682	756		
4	*petB*	LSC	6	761	657		
5	*petD*	LSC	9	750	474		
6	*rpl16*	LSC	8	1011	403		
7	*rpl2*	IR	391	673	434		
8	*rpoC1*	LSC	435	749	1620		
9	*rps12* *	LSC	114	-	232	543	26
10	*rps16*	LSC	39	899	228		
11	*trnA-UGC*	IR	38	814	35		
12	*trnG-UCC*	LSC	23	698	48		
13	*trnI-GAU*	IR	42	949	35		
14	*trnK-UUU*	LSC	37	2516	35		
15	*trnL-UAA*	LSC	37	554	50		
16	*trnV-UAC*	LSC	39	601	37		
17	*ycf3*	LSC	126	723	228	766	153

* *rps12* is a trans-spliced gene, of which two 3′ end residues are located in the IR region and the 5′ end in the LSC region.

**Table 4:** Codon usage in the *Sanguisorba officinalis* chloroplast genomes. RSCU: Relative Synonymous Codon Usage.

Amino Acid	Codon	Count	RSCU	tRNA	Amino Acid	Codon	Count	RSCU	tRNA
Phe	UUU	899	1.38		Tyr	UAU	682	1.61	
Phe	UUC	401	0.62	*trnF-GAA*	Tyr	UAC	165	0.39	*trnY-GUA*
Leu	UUA	810	2.03	*trnL-UAA*	Stop	UAA	43	1.65	
Leu	UUG	466	1.17	*trnL-CAA*	Stop	UAG	20	0.77	
Leu	CUU	503	1.26		His	CAU	403	1.51	
Leu	CUC	148	0.37		His	CAC	132	0.49	*trnH-GUG*
Leu	CUA	306	0.77	*trnL-UAG*	Gln	CAA	616	1.53	*trnQ-UUG*
Leu	CUG	156	0.39		Gln	CAG	191	0.47	
Ile	AUU	983	1.5		Asn	AAU	825	1.53	
Ile	AUC	369	0.56	*trnI-GAU*	Asn	AAC	256	0.47	*trnN-GUU*
Ile	AUA	614	0.94		Lys	AAA	925	1.54	*trnK-UUU*
Met	AUG	531	1	*trnfM-CAU*, *trnI-CAU*, *trnM-CAU*	Lys	AAG	280	0.46	
Val	GUU	471	1.48		Asp	GAU	712	1.62	
Val	GUC	152	0.48	*trnV-GAC*	Asp	GAC	168	0.38	*trnD-GUC*
Val	GUA	474	1.48	*trnV-UAC*	Glu	GAA	904	1.52	*trnE-UUC*
Val	GUG	180	0.56		Glu	GAG	287	0.48	
Ser	UCU	469	1.69		Cys	UGU	204	1.56	
Ser	UCC	263	0.95	*trnS-GGA*	Cys	UGC	57	0.44	*trnC-GCA*
Ser	UCA	306	1.1	*trnS-UGA*	Stop	UGA	15	0.58	
Ser	UCG	171	0.61		Trp	UGG	396	1	*trnW-CCA*
Pro	CCU	352	1.47		Arg	CGU	307	1.36	*trnR-ACG*
Pro	CCC	198	0.83		Arg	CGC	95	0.42	
Pro	CCA	257	1.08	*trnP-UGG*	Arg	CGA	312	1.38	
Pro	CCG	149	0.62		Arg	CGG	103	0.46	
Thr	ACU	465	1.59		Ser	AGU	349	1.25	
Thr	ACC	224	0.76	*trnT-GGU*	Ser	AGC	111	0.4	*trnS-GCU*
Thr	ACA	348	1.19	*trnT-UGU*	Arg	AGA	391	1.74	*trnR-UCU*
Thr	ACG	135	0.46		Arg	AGG	144	0.64	
Ala	GCU	576	1.79		Gly	GGU	524	1.32	
Ala	GCC	201	0.63		Gly	GGC	192	0.48	*trnG-GCC*
Ala	GCA	348	1.08	*trnA-UGC*	Gly	GGA	568	1.43	*trnG-UCC*
Ala	GCG	161	0.5		Gly	GGG	305	0.77	
Average # codons = 22,768									

**Table 5:** Types and numbers of SSRs found in the four *Sanguisorba* chloroplast genomes.

SSR Type	Repeat Unit	Number			
		*S. officinalis*	*S. filiformis*	*S. stipulata*	*S. tenuifolia* var. *alba*
Mono	A/T	55	56	68	53
	C/G	4	3	1	2
Di	AT/AT	11	9	8	11
	AG/CT	1	1	1	1
Tri	AAT/ATT	3	4	3	4
Tetra	AAAT/ATTT	4	3	5	4
	AAAG/CTTT	1	1	1	1
	ACAT/ATGT	1	1	1	1
	AGAT/ATCT	1	1	1	1
	AATT/AATT	0	1	1	0
Penta	AAATT/AATTT	1	0	0	1
Hexa	AAAGGG/CCCTTT	0	2	0	0
	AAAATC/ATTTTG	0	0	1	0
Total		82	82	91	79

## Supplementary Materials

Supplementary materials are available online. Table S1. The length of exons and introns in genes with introns in the *Sanguisorba filiformis* chloroplast genome. Table S2. The length of exons and introns in genes with introns in the *Sanguisorba stipulata* chloroplast genome. Table S3. The length of exons and introns in genes with introns in the *Sanguisorba tenuifolia* var. *alba* chloroplast genome. Table S4. Codon usage in the *Sanguisorba filiformis* chloroplast genome. Table S5. Codon usage in the *Sanguisorba stipulata* chloroplast genomes. Table S6. Codon usage in the *Sanguisorba tenuifolia* var. *alba* chloroplast genomes. Figure S1. Gene map of *Sanguisorba filiformis* chloroplast genome. Genes shown inside the circle are transcribed clockwise, and those outside are counterclockwise. Genes in different functional groups are color-coded. Figure S2. Gene map of *Sanguisorba stipulata* chloroplast genome. Genes shown inside the circle are transcribed clockwise, and those outside are counterclockwise. Genes in different functional groups are color-coded. Figure S3. Gene map of *Sanguisorba tenuifolia* var. *alba* chloroplast genom. Genes shown inside the circle are transcribed clockwise, and those outside are counterclockwise. Genes in different functional groups are color-coded. Figure S4. Phylogenetic relationships of the four *Sanguisorba* species constructed by protein coding genes using the maximum parsimony (MP) method. *Fragaria chiloensis* was set as the outgroup.
