# Supplementary material for: Complete Chloroplast Genomes from Sanguisorba: Identity and Variation Among Four Species
Source: Molecules. 2018 Aug 24;23(9):2137. doi: 10.3390/molecules23092137 (PMC6225366; doi:10.3390/molecules23092137)
Supplement: Supplementary file 1 [file molecules-23-02137-s001.zip › sup/Figure S3.docx]

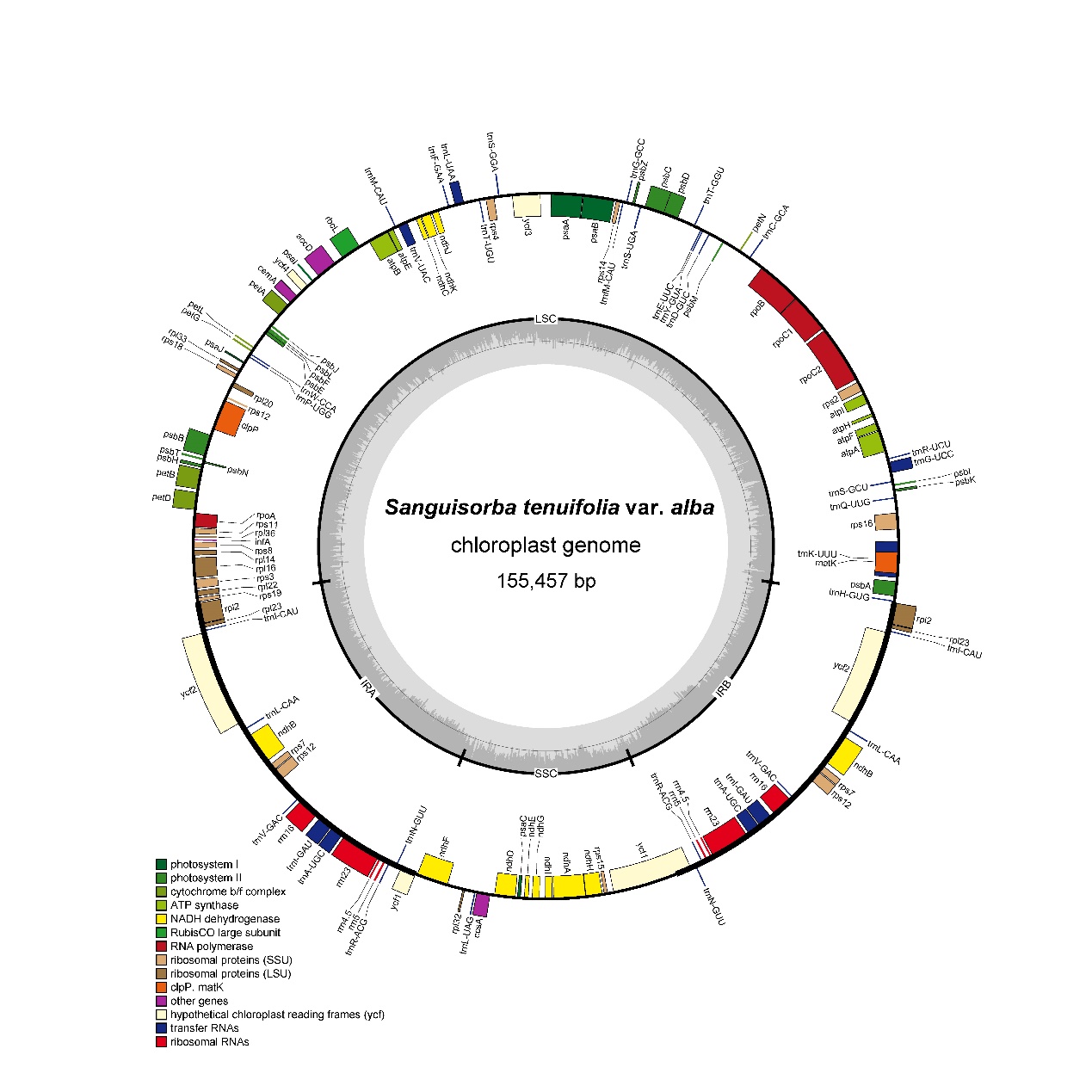


Figure S3. Gene map of *Sanguisorba tenuifolia* var. *alba* chloroplast genom. Genes shown inside the circle are transcribed clockwise, and those outside are counterclockwise. Genes in different functional groups are color-coded.
