# Supplementary material for: Complete Chloroplast Genomes from Sanguisorba: Identity and Variation Among Four Species
Source: Molecules. 2018 Aug 24;23(9):2137. doi: 10.3390/molecules23092137 (PMC6225366; doi:10.3390/molecules23092137)
Supplement: Supplementary file 1 [file molecules-23-02137-s001.zip › sup/Figure S4.docx]

Figure S4. Phylogenetic relationships of the four *Sanguisorba* species constructed by protein coding genes using the maximum parsimony (MP) method. *Fragaria chiloensis* was set as the outgroup.
