# Supplementary material for: Complete Chloroplast Genomes from Sanguisorba: Identity and Variation Among Four Species
Source: Molecules. 2018 Aug 24;23(9):2137. doi: 10.3390/molecules23092137 (PMC6225366; doi:10.3390/molecules23092137)
Supplement: Supplementary file 1 [file molecules-23-02137-s001.zip › sup/Table S1.docx]

Table S1 The length of exons and introns in genes with introns in the *Sanguisorba filiformis* chloroplast genomes.

| No. | Gene | Location | Exon I (bp) | Intron I (bp) | Exon II (bp) | Intron I (bp) | Exon III (bp) |
| --- | --- | --- | --- | --- | --- | --- | --- |
| 1 | *clpP* | LSC | 69 | 1876 | 291 | 657 | 228 |
| 2 | *ndhA* | SSC | 563 | 192 | 541 |  |  |
| 3 | *ndhB* | IR | 777 | 676 | 756 |  |  |
| 4 | *petB* | LSC | 6 | 753 | 657 |  |  |
| 5 | *petD* | LSC | 9 | 725 | 474 |  |  |
| 6 | *rpl16* | LSC | 8 | 953 | 403 |  |  |
| 7 | *rpl2* | IR | 391 | 673 | 434 |  |  |
| 8 | *rpoC1* | LSC | 435 | 758 | 1620 |  |  |
| 9 | *rps12** | LSC | 114 | - | 232 | 543 | 26 |
| 10 | *rps16* | LSC | 39 | 902 | 228 |  |  |
| 11 | *trnA-UGC* | IR | 38 | 811 | 35 |  |  |
| 12 | *trnG-UCC* | LSC | 23 | 698 | 48 |  |  |
| 13 | *trnI-GAU* | IR | 42 | 950 | 35 |  |  |
| 14 | *trnK-UUU* | LSC | 37 | 2508 | 35 |  |  |
| 15 | *trnL-UAA* | LSC | 37 | 554 | 50 |  |  |
| 16 | *trnV-UAC* | LSC | 39 | 601 | 37 |  |  |
| 17 | *ycf3* | LSC | 126 | 719 | 228 | 772 | 153 |

*The rps12 is a trans-spliced gene, which two 3’ end residues located in the IR region and 5’ end in the LSC region.
