# Supplementary material for: Complete Chloroplast Genomes from Sanguisorba: Identity and Variation Among Four Species
Source: Molecules. 2018 Aug 24;23(9):2137. doi: 10.3390/molecules23092137 (PMC6225366; doi:10.3390/molecules23092137)
Supplement: Supplementary file 1 [file molecules-23-02137-s001.zip › sup/Table S4.docx]

Table S4 Codon usage in the *Sanguisorba filiformis* chloroplast genomes.

| Amino Acid | Codon | Count | RSCU | tRNA | Amino Acid | Codon | Count | RSCU | tRNA |
| --- | --- | --- | --- | --- | --- | --- | --- | --- | --- |
| Phe | UUU | 905 | 1.39 |  | Tyr | UAU | 681 | 1.61 |  |
| Phe | UUC | 400 | 0.61 | *trnF-GAA* | Tyr | UAC | 165 | 0.39 | *trnY-GUA* |
| Leu | UUA | 811 | 2.03 | *trnL-UAA* | Stop | UAA | 46 | 1.77 |  |
| Leu | UUG | 472 | 1.18 | *trnL-CAA* | Stop | UAG | 19 | 0.73 |  |
| Leu | CUU | 506 | 1.26 |  | His | CAU | 400 | 1.52 |  |
| Leu | CUC | 147 | 0.37 |  | His | CAC | 128 | 0.48 | *trnH-GUG* |
| Leu | CUA | 308 | 0.77 | *trnL-UAG* | Gln | CAA | 611 | 1.5 | *trnQ-UUG* |
| Leu | CUG | 156 | 0.39 |  | Gln | CAG | 201 | 0.5 |  |
| Ile | AUU | 995 | 1.52 |  | Asn | AAU | 828 | 1.52 |  |
| Ile | AUC | 367 | 0.56 | *trnI-GAU* | Asn | AAC | 260 | 0.48 | *trnN-GUU* |
| Ile | AUA | 606 | 0.92 |  | Lys | AAA | 928 | 1.54 | *trnK-UUU* |
| Met | AUG | 521 | 1 | *trnfM-CAU, trnI-CAU,*  *trnM-CAU* | Lys | AAG | 274 | 0.46 |  |
| Val | GUU | 473 | 1.48 |  | Asp | GAU | 708 | 1.62 |  |
| Val | GUC | 151 | 0.47 | *trnV-GAC* | Asp | GAC | 165 | 0.38 | *trnD-GUC* |
| Val | GUA | 480 | 1.5 | *trnV-UAC* | Glu | GAA | 898 | 1.51 | *trnE-UUC* |
| Val | GUG | 173 | 0.54 |  | Glu | GAG | 292 | 0.49 |  |
| Ser | UCU | 462 | 1.66 |  | Cys | UGU | 201 | 1.55 |  |
| Ser | UCC | 269 | 0.97 | *trnS-GGA* | Cys | UGC | 59 | 0.45 | *trnC-GCA* |
| Ser | UCA | 308 | 1.11 | *trnS-UGA* | Stop | UGA | 13 | 0.5 |  |
| Ser | UCG | 169 | 0.61 |  | Trp | UGG | 391 | 1 | *trnW-CCA* |
| Pro | CCU | 352 | 1.48 |  | Arg | CGU | 308 | 1.36 | *trnR-ACG* |
| Pro | CCC | 200 | 0.84 |  | Arg | CGC | 95 | 0.42 |  |
| Pro | CCA | 253 | 1.06 | *trnP-UGG* | Arg | CGA | 316 | 1.4 |  |
| Pro | CCG | 148 | 0.62 |  | Arg | CGG | 105 | 0.46 |  |
| Thr | ACU | 466 | 1.59 |  | Ser | AGU | 351 | 1.26 |  |
| Thr | ACC | 216 | 0.74 | *trnT-GGU* | Ser | AGC | 109 | 0.39 | *trnS-GCU* |
| Thr | ACA | 357 | 1.22 | *trnT-UGU* | Arg | AGA | 389 | 1.72 | *trnR-UCU* |
| Thr | ACG | 132 | 0.45 |  | Arg | AGG | 142 | 0.63 |  |
| Ala | GCU | 577 | 1.79 |  | Gly | GGU | 529 | 1.33 |  |
| Ala | GCC | 202 | 0.63 |  | Gly | GGC | 189 | 0.48 | *trnG-GCC* |
| Ala | GCA | 350 | 1.09 | *trnA-UGC* | Gly | GGA | 568 | 1.43 | *trnG-UCC* |
| Ala | GCG | 158 | 0.49 |  | Gly | GGG | 301 | 0.76 |  |
| Average# codons=22760 | | | | | | | | | |

RSCU: Relative Synonymous Codon Usage.
