# Supplementary material for: Complete Chloroplast Genomes from Sanguisorba: Identity and Variation Among Four Species
Source: Molecules. 2018 Aug 24;23(9):2137. doi: 10.3390/molecules23092137 (PMC6225366; doi:10.3390/molecules23092137)
Supplement: Supplementary file 1 [file molecules-23-02137-s001.zip › sup/Table S5.docx]

Table S5 Codon usage in the *Sanguisorba stipulata* chloroplast genomes.

| Amino Acid | Codon | Count | RSCU | tRNA | Amino Acid | Codon | Count | RSCU | tRNA |
| --- | --- | --- | --- | --- | --- | --- | --- | --- | --- |
| Phe | UUU | 899 | 1.38 |  | Tyr | UAU | 683 | 1.61 |  |
| Phe | UUC | 401 | 0.62 | *trnF-GAA* | Tyr | UAC | 163 | 0.39 | *trnY-GUA* |
| Leu | UUA | 809 | 2.03 | *trnL-UAA* | Stop | UAA | 44 | 1.69 |  |
| Leu | UUG | 468 | 1.18 | *trnL-CAA* | Stop | UAG | 20 | 0.77 |  |
| Leu | CUU | 501 | 1.26 |  | His | CAU | 404 | 1.51 |  |
| Leu | CUC | 149 | 0.37 |  | His | CAC | 131 | 0.49 | *trnH-GUG* |
| Leu | CUA | 300 | 0.75 | *trnL-UAG* | Gln | CAA | 618 | 1.53 | *trnQ-UUG* |
| Leu | CUG | 160 | 0.4 |  | Gln | CAG | 190 | 0.47 |  |
| Ile | AUU | 988 | 1.51 |  | Asn | AAU | 825 | 1.52 |  |
| Ile | AUC | 367 | 0.56 | *trnI-GAU* | Asn | AAC | 259 | 0.48 | *trnN-GUU* |
| Ile | AUA | 613 | 0.93 |  | Lys | AAA | 931 | 1.54 | *trnK-UUU* |
| Met | AUG | 529 | 1 | *trnfM-CAU, trnI-CAU,*  *trnM-CAU* | Lys | AAG | 277 | 0.46 |  |
| Val | GUU | 474 | 1.48 |  | Asp | GAU | 713 | 1.62 |  |
| Val | GUC | 153 | 0.48 | *trnV-GAC* | Asp | GAC | 166 | 0.38 | *trnD-GUC* |
| Val | GUA | 478 | 1.49 | *trnV-UAC* | Glu | GAA | 903 | 1.52 | *trnE-UUC* |
| Val | GUG | 178 | 0.55 |  | Glu | GAG | 288 | 0.48 |  |
| Ser | UCU | 465 | 1.67 |  | Cys | UGU | 207 | 1.58 |  |
| Ser | UCC | 271 | 0.98 | *trnS-GGA* | Cys | UGC | 55 | 0.42 | *trnC-GCA* |
| Ser | UCA | 303 | 1.09 | *trnS-UGA* | Stop | UGA | 14 | 0.54 |  |
| Ser | UCG | 170 | 0.61 |  | Trp | UGG | 395 | 1 | *trnW-CCA* |
| Pro | CCU | 349 | 1.46 |  | Arg | CGU | 306 | 1.36 | *trnR-ACG* |
| Pro | CCC | 199 | 0.83 |  | Arg | CGC | 97 | 0.43 |  |
| Pro | CCA | 258 | 1.08 | *trnP-UGG* | Arg | CGA | 306 | 1.36 |  |
| Pro | CCG | 149 | 0.62 |  | Arg | CGG | 104 | 0.46 |  |
| Thr | ACU | 462 | 1.58 |  | Ser | AGU | 349 | 1.26 |  |
| Thr | ACC | 220 | 0.75 | *trnT-GGU* | Ser | AGC | 109 | 0.39 | *trnS-GCU* |
| Thr | ACA | 352 | 1.21 | *trnT-UGU* | Arg | AGA | 392 | 1.74 | *trnR-UCU* |
| Thr | ACG | 133 | 0.46 |  | Arg | AGG | 146 | 0.65 |  |
| Ala | GCU | 577 | 1.79 |  | Gly | GGU | 527 | 1.33 |  |
| Ala | GCC | 203 | 0.63 |  | Gly | GGC | 188 | 0.47 | *trnG-GCC* |
| Ala | GCA | 345 | 1.07 | *trnA-UGC* | Gly | GGA | 568 | 1.43 | *trnG-UCC* |
| Ala | GCG | 161 | 0.5 |  | Gly | GGG | 304 | 0.77 |  |
| Average# codons=22766 | | | | | | | | | |

RSCU: Relative Synonymous Codon Usage.
