# Supplementary material for: Complete Chloroplast Genomes from Sanguisorba: Identity and Variation Among Four Species
Source: Molecules. 2018 Aug 24;23(9):2137. doi: 10.3390/molecules23092137 (PMC6225366; doi:10.3390/molecules23092137)
Supplement: Supplementary file 1 [file molecules-23-02137-s001.zip › sup/Table S6.docx]

Table S6 Codon usage in the *Sanguisorba tenuifolia* var. *alba* chloroplast genomes.

| Amino Acid | Codon | Count | RSCU | tRNA | Amino Acid | Codon | Count | RSCU | tRNA |
| --- | --- | --- | --- | --- | --- | --- | --- | --- | --- |
| Phe | UUU | 899 | 1.38 |  | Tyr | UAU | 682 | 1.61 |  |
| Phe | UUC | 401 | 0.62 | *trnF-GAA* | Tyr | UAC | 165 | 0.39 | *trnY-GUA* |
| Leu | UUA | 810 | 2.03 | *trnL-UAA* | Stop | UAA | 43 | 1.65 |  |
| Leu | UUG | 467 | 1.17 | *trnL-CAA* | Stop | UAG | 20 | 0.77 |  |
| Leu | CUU | 503 | 1.26 |  | His | CAU | 403 | 1.51 |  |
| Leu | CUC | 148 | 0.37 |  | His | CAC | 132 | 0.49 | *trnH-GUG* |
| Leu | CUA | 306 | 0.77 | *trnL-UAG* | Gln | CAA | 616 | 1.53 | *trnQ-UUG* |
| Leu | CUG | 156 | 0.39 |  | Gln | CAG | 191 | 0.47 |  |
| Ile | AUU | 984 | 1.5 |  | Asn | AAU | 825 | 1.52 |  |
| Ile | AUC | 370 | 0.56 | *trnI-GAU* | Asn | AAC | 257 | 0.48 | *trnN-GUU* |
| Ile | AUA | 612 | 0.93 |  | Lys | AAA | 924 | 1.53 | *trnK-UUU* |
| Met | AUG | 530 | 1 | *trnfM-CAU, trnI-CAU,*  *trnM-CAU* | Lys | AAG | 280 | 0.47 |  |
| Val | GUU | 471 | 1.47 |  | Asp | GAU | 712 | 1.62 |  |
| Val | GUC | 152 | 0.48 | *trnV-GAC* | Asp | GAC | 168 | 0.38 | *trnD-GUC* |
| Val | GUA | 475 | 1.49 | *trnV-UAC* | Glu | GAA | 904 | 1.52 | *trnE-UUC* |
| Val | GUG | 180 | 0.56 |  | Glu | GAG | 287 | 0.48 |  |
| Ser | UCU | 470 | 1.69 |  | Cys | UGU | 204 | 1.56 |  |
| Ser | UCC | 263 | 0.95 | *trnS-GGA* | Cys | UGC | 57 | 0.44 | *trnC-GCA* |
| Ser | UCA | 306 | 1.1 | *trnS-UGA* | Stop | UGA | 15 | 0.58 |  |
| Ser | UCG | 170 | 0.61 |  | Trp | UGG | 395 | 1 | *trnW-CCA* |
| Pro | CCU | 352 | 1.47 |  | Arg | CGU | 307 | 1.36 | *trnR-ACG* |
| Pro | CCC | 198 | 0.83 |  | Arg | CGC | 95 | 0.42 |  |
| Pro | CCA | 258 | 1.08 | *trnP-UGG* | Arg | CGA | 312 | 1.38 |  |
| Pro | CCG | 148 | 0.62 |  | Arg | CGG | 103 | 0.46 |  |
| Thr | ACU | 465 | 1.59 |  | Ser | AGU | 349 | 1.25 |  |
| Thr | ACC | 224 | 0.76 | *trnT-GGU* | Ser | AGC | 111 | 0.4 | *trnS-GCU* |
| Thr | ACA | 348 | 1.19 | *trnT-UGU* | Arg | AGA | 392 | 1.74 | *trnR-UCU* |
| Thr | ACG | 135 | 0.46 |  | Arg | AGG | 144 | 0.64 |  |
| Ala | GCU | 576 | 1.79 |  | Gly | GGU | 524 | 1.32 |  |
| Ala | GCC | 201 | 0.63 |  | Gly | GGC | 192 | 0.48 | *trnG-GCC* |
| Ala | GCA | 348 | 1.08 | *trnA-UGC* | Gly | GGA | 567 | 1.43 | *trnG-UCC* |
| Ala | GCG | 161 | 0.5 |  | Gly | GGG | 305 | 0.77 |  |
| Average# codons=22768 | | | | | | | | | |

RSCU: Relative Synonymous Codon Usage.
